# Feasibility, impact, and priority of key strategies to enhance diverse and inclusive training programs in clinical and translational research: A mixed methods study

**DOI:** 10.1017/cts.2022.452

**Published:** 2022-08-25

**Authors:** Jennifer A. Campbell, Rebekah J. Walker, Aprill Z. Dawson, Mukoso N. Ozieh, Susanne Schmidt, L. Aubree Shay, Joni S. Williams, Shane A. Phillips, Leonard E. Egede

**Affiliations:** 1 Division of General Internal Medicine, Department of Medicine, Medical College of Wisconsin, Milwaukee, WI, USA; 2 Center for Advancing Population Science, Medical College of Wisconsin, Milwaukee, WI, USA; 3 Department of Population Health Sciences UT Health San Antonio, San Antonio, TX, USA; 4 Department of Health Promotion & Behavioral Sciences UT Health School of Public Health in San Antonio, San Antonio, TX, USA; 5 Department of Physical Therapy, College of Applied Health Sciences, University of Illinois at Chicago, Chicago, IL, USA

**Keywords:** Diversity, equity, inclusion, clinical and translational research, mixed methods

## Abstract

**Background:**

Enhancing diversity in the scientific workforce is a long-standing issue. This study uses mixed methods to understand the feasibility, impact, and priority of six key strategies to promote diverse and inclusive training and contextualize the six key strategies across Clinical and Translational Science Awards (CTSAs) Program Institutions.

**Methods:**

Four breakout sessions were held at the NCATS 2020 CTSA Program annual meeting focused on diversity, equity, and inclusion (DEI) efforts. This paper focuses on the breakout session for Enhancing DEI in Translational Science Training Programs. Data were analyzed using a mixed methods convergent approach. The quantitative strand includes the online polling results. The qualitative strand includes the breakout session and the chat box in response to the training presentation.

**Results:**

Across feasibility, impact, and priority questions, *prioritizing representation* ranked number 1. *Building partnerships* ranked number 2 in feasibility and priority, while making it personal ranked number 2 for impact. Across each strategy, rankings supported the qualitative data findings in feasibility through shared experiences, impact in the ability to increase DEI, and priority rankings in comparison to the other strategies. No divergence was found across quantitative and qualitative data findings.

**Conclusion:**

Findings provide robust support for prioritizing representation as a number one strategy to focus on in training programs. Specifically, this strategy can be operationalized through integration of community representation, diversity advocates, and adopting a holistic approach to recruiting a diverse cadre of scholars into translational science training programs at the national level across CTSAs.

## Introduction

The confluence of the COVID-19 pandemic, social unrest, and the explicit manifestation of racism against communities of color, has led to the adoption of structural racism in the daily vernacular of mainstream media and scientific discourse alike [1-6]. The national foregrounding of structural racism since Spring 2020 has fostered strategic realignment across societal sectors to dismantle the mechanisms that reinforce structural racism [5,7]. These strategies are being unified by the scientific community in multiple ways. First, by naming and acknowledging what has long been experienced by communities of color. Second, by a call to generate research that identifies and addresses mechanisms of structural racism that have fundamentally suppressed health and wellbeing, and third, by fostering diversity, equity, and inclusion (DEI), with an emphasis on training in the health sector [7].

The lack of diverse representation within health care and the scientific community is a longstanding issue [8-11]. Recently highlighted in the call for revaluation of DEI efforts within the national consortium of the Clinical and Translational Science Awards (CTSAs), Boulware and colleagues emphasize the need for achievable goals and recommended strategies to increase DEI across leadership, training, research, and clinical trials recruitment/participation [12]. Specifically, to diversify the healthcare workforce, clinician investigators, and the scientific community at large, prioritization is needed in the development of trainees across stages of educational and scientific development [12]. To effectively achieve this, it is recommended that partnerships be developed that provide exposure and training to health equity research early on in educational pathways, support be provided for mentors, regardless of social background, who are training ethnically and culturally diverse scholars, and program culture shifts toward emphasizing the lived experiences throughout the training process [12].

To effect this change, reorganization of programmatic elements emphasizing DEI in training is greatly needed to address the long-standing gaps that have limited diversity in clinical and translational research. The University of Rochester Center for Leading Innovation and Collaboration (CLIC), the coordinating center the CTSA program, developed *From Insights to Action: Enriching the Clinical Research Workforce by Developing Diverse and Inclusive Career Programs,* outlined six key strategies that can be used as a guide for CTSA programs across the nation [13]. These key strategies include: 1) prioritizing representation, 2) building partnerships, 3) designing program structure, 4) making it personal, 5) improving through feedback, and 6) winning endorsements. With the key strategies identified, moving strategy to action will require operationalization with strategic goals anchored in training programs across CTSA programs [13].

Using a mixed methods approach, this paper presents data from the NCATS 2020 CTSA Program annual meeting focused on how training efforts across CTSA program hubs can operationalize the six key strategies developed and presented by CLIC [13]. The primary aims of this paper include the following: 1) To understand the feasibility, impact, and priority of six key strategies to promote diverse and inclusive training across CTSA Program institutions. 2) To contextualize the six key strategies across CTSA Program Institutions. 3) To compare qualitative and quantitative strands of data to gain a more in-depth understanding of priorities and context of the six key strategies to promote diverse and inclusive career programs across CTSA program institutions.

## Materials and Methods

### 2020 Fall CTSA Program Meeting Overview

This study analyzed data from the NCATS 2020 annual meeting representing approximately 60 CTSA Program Institutions in the United States. This meeting was held virtually. The meeting agenda was established by the steering committee and the 2020 meeting agenda was developed through feedback from consortium members, wherein DEI emerged as a priority. Meeting attendees participated in this conference with the understanding that results of the meeting would be analyzed and disseminated scientifically.

Four breakout sessions were developed that included Workforce Development, CTSA Consortium Leadership, Disparities/Health Equity Research, and Clinical Trials Participation. At each breakout session, priorities identified from the initial poll were included with key leaders providing a presentation followed by an additional poll to assess the feasibility, impact, and priority of the key areas. This paper focuses on the breakout session for Enhancing Diversity and Inclusion in Translational Science Training Programs. This training session used as its framework *From Insights to Action: Enriching the Clinical Research Workforce by Developing Diverse and Inclusive Career Programs*, developed by the CTSA coordinating center, CLIC, to outline six key strategies. These strategies included: 1. Prioritizing representation; 2. Building partnerships; 3. Designing program structure; 4. Making it personal; 5. Improving thorough feedback; and 6. Winning endorsement. Attendees in this breakout session were encouraged to provide feedback around incorporating DEI into current strategies, dissemination of best practices, and specific for clinical and translational science and research pre- and post-doctoral scholars (TL1s), discussed the structure needed to reduce disparities. Respondents completing the poll totaled 231 (29% response rate), representing 54 CTSA hubs out of 64 CTSA programs. The professional representation of the respondents included executive directors/administrators (15%), principal investigators (13%), and other (50%). A total of 94% of respondents endorsed DEI as being extremely or very important with 86% also indicating they are extremely or very committed to improve DEI efforts.

Results from this session were analyzed using a mixed methods convergent approach. This approach to analysis is appropriate for the collection of parallel, complimentary data, using quantitative and qualitative methods [14]. The intention of this design is to harness the strength of two complimentary methods for data collection and to gain in depth understanding of a given research question. The four recommended phases for a mixed methods convergent design were followed. These include: Phase 1, data collection for both qualitative and quantitative strands collected separately. Phase 2, separate analysis for each quantitative and qualitative strand. Phase 3, merging or integration of the quantitative and qualitative data sets. Lastly, Phase 4, the interpretation of the integrated results including summarizing the findings and discussing the extent to which data from both strands converged, diverged, and the extent to which a more complete understanding was produced [14].

### Quantitative

The quantitative strand included the online polling results from the NCATS 2020 annual meeting. Data were collected electronically through an online polling platform, polleverywhere, set up, and managed by the conference. The online polling was done in real time. The poll asked during the breakout session came from three previously agreed upon areas that each of the leads of the four breakouts agreed to ask: 1) which strategies would be most feasible at your CTSA?, 2) which strategies will likely have the greatest impact? and 3) which strategies would you suggest giving the highest priority?). The breakout participants were then asked to rank the answers based on each of the questions focusing the feasibility, impact, and priority of each. There were 44 participants, 34 who completed responses for the quantitative strand in the training session, representing 77% engagement.

### Qualitative

The qualitative strand of this data included data from the breakout session on training and the chat box in response to the training presentation. The breakout sessions were transcribed, and a transcription of the chat box was provided by the polleverywhere platform following the conference.

### Statistical Analysis

For the quantitative strand, frequencies and ranking were analyzed through the online polling platform in real time and a summary of the polling report was provided at the close of the conference. For the qualitative strand, a thematic analysis approach was used to analyze both the breakout sessions and the chat box for the training session. This approach was chosen due to the nature of the data, which was to understand and identify meaning and phenomena as it relates to feasibility, impact, and priority for the six themes within the context of training efforts across CTSA program hubs. Each of the steps outlined by Ritchie et al. (2013), using Microsoft Word and Excel, were followed to analyze the interviews, 1) familiarization, 2) constructing initial thematic framework, 3) indexing and sorting, and 4) reviewing data extracts [15]. Worksheets for each of these steps were used to keep a record of topics and themes as they emerged. The thematic framework was developed according to the six strategies and initial familiarization with the data allowed for the data to be identified by theme to frame the findings. Integration of these two data sets included comparing qualitative and quantitative strands of data to gain a more in-depth understanding of priorities and context of six key strategies to promote diverse and inclusive career programs across CTSA program institutions. In addition, integration involved identifying areas of convergence or divergence across the two datasets.

## Results

### Quantitative

The quantitative aim of this analysis was to understand the feasibility, impact, and priority of the six key strategies to promote diverse and inclusive training across CTSA Program institutions from: Center for Leading Innovation and Collaboration (CLIC). From Insights to Action: a resource for hubs looking for ways to increase the diversity of their clinical science workforce [13]. Table 1 summarizes the key strategies along with their respective definitions. Table 2 shows the polling report ranking each area specific to Enhancing DEI in Translational Science Training Programs. For feasibility, prioritizing representation ranked number 1, building partnerships ranked number 2, making it personal ranked number 3, designing program structure ranked number 4, improving through feedback ranked number 5, and winning endorsement ranked number 6. For impact, prioritizing representation ranked number 1, making it personal ranked number 2, building partnerships ranked number 3, designing program structure ranked number 4, winning endorsement ranked number 5, and improving through feedback ranked number 6. For priority, prioritizing representation ranked number 1, building partnerships ranked number 2, designing program structure ranked number 4, winning endorsement ranked number 5, and improving through feedback ranked number 6.

**Table 1.** Key strategies to improve diversity equity and inclusion in clinical and translational training programs

Prioritizing Representation	Prioritize representation, holistically, at every stage of the career pathway.
Building Partnerships	Actively collaborate with departmental, institutional, and across-institutional leadership on common/aligned programmatic goals.
Designing Program Structure	Balance structured programmatic supports with space for scholar-led innovation.
Making it Personal	Value and nurture the whole scholar: past, present, and future.
Improving Through Feedback	Value and nurture the whole scholar: past, present, and future.
Winning Endorsement	An inclusive program seeks and sees advocates outside of its own leadership.

Source: Center for Leading Innovation and Collaboration (CLIC) From Insights To Action [13].

**Table 2.** Polling report – diversity equity and inclusion session breakout – training: ranking of feasibility, impact, and priority of six key strategies

Strategy	Feasibility	Impact	Priority
Prioritizing Representation	1	1	1
Building Partnerships	2	3	2
Making it Personal	3	2	3
Designing Program Structure	4	4	4
Improving Through Feedback	5	6	6
Winning Endorsement	6	5	5

### Qualitative

The aim of the qualitative strand was to contextualize the six key strategies across CTSA Program Institutions in the United States. Table 3 illustrates the chat box responses, aligned to each of the six key strategies. Below are quotes that support each of the six key strategies from the chat box.

**Table 3.** Chat box qualitative results from Diversity Equity and Inclusion Training breakout session mapped to Key DEI Strategies

Strategy	Qualitative Results*
Prioritizing Representation	Interview process - use P.A.U.S.E. (**P**ay attention; **A**cknowledge your assumptions; **U**nderstand your perspective; **S**eek different perspectives; **E**xamine your options and make a decision) to try to reduce bias
	It’s not about ability, it’s about access. We need better access to our programs and encourage BIPOC to apply and mentor them through the process
	For TL1 [Clinical and Translational Science Fellowship], we interview anyone from an underrepresented or disadvantaged background
	Summer research internship programs help us identify a diverse population. We follow that up with yearlong undergraduate programs; we partner them with mentors and move them into the predoc TL1 then the post doc Tl1 and then a k.
	A holistic review is important and department admissions could mirror that.
Building Partnerships	RCMI institutions have a lot in common with CTSA institutions - partnering in meaningful ways (like research partnerships) with RCMI institutions is a great way to learn more about common interests and to share opportunities across institutions.
	We should be champions of dissemination and, while we build partnerships across programs in our institutions, we disseminate to those communities.
	A challenge to TL1 programs is that we depend on our institutional programs to admit predocs and hire postdocs, and we select trainees from that local pool. We need to partner with all of them.
	While our program is small and multi-institutional, we have addressed some of these concerns by promoting 1-cross institutional mentorship, 2-facilitating movement of predocs from one partner institution to TL1 postdocs at another in our Hub, 3-retaining TL1 postdocs, with evolving mentorship plans/teams in our KL2.
	We are working with a vocational school to diversify our pool of coordinators.
	It’s hard to diversify if you have no community connections.
Designing Program Structure	Train “non diverse” mentors to be effective mentors for diverse students
	Building pathways…
	We need to advance culturally aware mentorship education
	When selecting trainees think about creating a community of learners as part of the decision making
	Include cultural competence as part of curriculum
	Isolation is a significant barrier - we need effective communities of students with near peer and peer to peer mentoring
	What helps TL1 programs develop more diverse trainee communities? Prioritize diversity explicitly, create a culture where diversity and anti-racism are explicit and publicly endorsed priorities, train mentors to better understand their roles, meet the needs of trainees once they’re in the program, celebrate success, network, develop meaningful research programs that interest a wide range of trainees, show caring, welcoming, understanding…listen to trainees.
	For our TL1, we have a track in CTS in the graduate school that results in a PhD. This has proved very popular with outstanding URM students (>50% of the matriculants are URMs). We think that this in part reflects that many are interested in more late-stage translation that can directly impact communities, among other factors.
	We treat scores and reviews as advisory. There is a false precision in our scoring process, and we give too much weight to this. Really, what is the difference in scores of 2.5 and 2.8? We take diversity and the holistic view into account when we make appointments.
Making It Personal	We think it’s important to have multiple “on ramps” and to build in second chances for those who might have missed the earlier, perhaps most usual, on ramps to research career development
	We start at the pre-application stage in order to give individualized feedback to scholars (and their mentors) in navigating the process and in strengthening their applications, so that they do not “fall off” along the application path
	You do not need to BE diverse to be effective in advocating and implementing effective programs
	I offer to help everyone with their application. I make a personal offer to people who are underrepresented.
	AAMC meeting this week, I listened to the stories behind black doctor’s CVs. so powerful. African American male who was an orphan or another whose father was shot and killed. The struggles that they had to get into medical schools because they didn’t fit the “profile”
Improving Through Feedback	An effective IDP “process” should include feedback. as clinicians we are trained to provide feedback to residents and students. As researchers this training is less common
	Helping mentors and mentee to effectively (and regularly) discussed IDPs as a process is an important part of mentorship education.
	Students are not fans of IDPs in general - this makes me think we are not effectively using the IDP process to provide feedback
	I think a first step is showing the trainees we actually review the IDPs and take them seriously. I will bring up the IDP and review milestones/goals during 1 on 1 sessions with trainees
	Part of the issue is their hesitancy is sharing with their mentor.
	I am not the TL1 fellows’ official research mentor so I try to provide a space where they can safely speak about inclusion and diversity issues and any problems they are having in this regard with their research team
	We have added a DEI position to our education leadership. one of his roles is a safe space for students. most common conversations focus on supporting students experiencing microaggressions
Winning Endorsement	URM would benefit from more sponsorship - beyond mentorship.
	Mentorship. sponsorship and coaching…
	And helping these networks of mentors work as a collective is a whole interesting area for mentorship education
	Our TL1 postdoc mentoring teams include a mentor from another hub or associated institution - helps with networking and building collaborators.

*Response presented as written.

Acronyms Defined: AAMC [Association of American Medical Colleges]; BIPOC [Black Indigenous People of Color]; CTS [Clinical and Translational Science]; CTSA [Clinical and Translational Science Awards]; CV [Curriculum Vitae]; DEI [Diversity Equity and Inclusion]; IDP [Individual Development Plan]; KL2 [Mentored Career Development Award]; RCMI [Research Centers in Minority Institutions]; TL1 [Clinical and Translational Science Fellowship]; URM [Underrepresented Minority].

### Respondent Summaries by Strategy

*Prioritizing Representation* was characterized through access and the interview process: “It’s not about ability, it’s about access. We need better access to our programs and encourage BIPOC (black, Indigenous, and people of color) to apply and mentor them through the process.” “We interview anyone from an underrepresented or disadvantaged background.” “Summer research internship programs help us identify a diverse population. We follow that up with yearlong undergraduate programs; we partner them with mentors and move them into the predoc TL1 then the post doc TL1 and then a K.”

*Building Partnerships* was characterized by actively collaborating with departmental, institutional, and across-institutional leadership on common/aligned programmatic goals: “RCMI institutions have a lot in common with CTSA institutions - partnering in meaningful ways (like research partnerships) with RCMI [*Research Centers in Minority Institutions*] institutions is a great way to learn more about common interests and to share opportunities across institutions.” “A challenge to TL1 programs is that we depend on our institutional programs to admit predocs and hire postdocs, and we select trainees from that local pool. We need to partner with all of them.” “While our program is small and multi-institutional, we have addressed some of these concerns by promoting 1-cross institutional mentorship, 2-facilitating movement of predocs from one partner institution to TL1 postdocs at another in our Hub, 3-retaining TL1 postdocs, with evolving mentorship plans/teams in our KL2.”

*Designing Program Structure* was largely reflected by discussion around the structure for adequate mentorship as well as training mentors who are not underrepresented in medicine to be effective at mentorship for diverse mentees: “[There is need to] train ‘non diverse’ mentors to be effective mentors for diverse students. We need to advance culturally aware mentorship education and include cultural competence as part of curriculum. It starts with addressing both conscious and unconscious bias.” “I love the group mentoring approach. One challenge with it is helping mentors and mentees to optimize the group mentoring experience. We cannot assume that we all know how to effectively lead a group of mentors…there are skills and tools.”

*Making it Personal* was found across the chat as participants discussed strategies used from the preapplication phase as well as the need to value and account for the scholars’ lived experience: “We start at the pre-application stage in order to give individualized feedback to scholars (and their mentors) in navigating the process and in strengthening their applications, so that they do not ‘fall off’ along the application path.” “I listened to the stories behind black doctors CVs. So powerful. African American male who was an orphan or another whose father was shot and killed. The struggles that they had to get into medical schools because they didn’t fit the ‘profile.’”

*Improving Through Feedback* was seen through respondents highlighting a particular mechanism for feedback: “An effective IDP [*Individual Development Plan*] ‘process’ should include feedback. As clinicians we are trained to provide feedback to residents and students. As researchers this training is less common.” “I think a first step is showing the trainees we actually review the IDPs and take them seriously. I will bring up the IDP and review milestones/goals during one-on-one sessions with trainees. Part of the issue is their hesitancy is sharing with their mentor.”

*Winning Endorsement* was reflected in conversations around mentorship and the need to build mentorship networks that extend beyond one’s home institution: “URM would benefit from more sponsorship - beyond mentorship.” “Our TL1 postdoc mentoring teams include a mentor from another hub or associated institution – helps with networking and building collaborators.”

### Integration of Quantitative and Qualitative Data Sets

Table 4 shows the joint display of the rankings of each area by feasibility, impact, and priority with the qualitative findings as well as the mixed methods comparison. The rankings of each strategy were compared to the qualitative data results to identify areas of similarity and dissimilarity. If similar, the data were considered to have converged. If dissimilar, the data were considered to have diverged. The convergent data analysis showed similarity across Prioritizing Representation, Building Partnerships, Making it Personal, Designing Program Structure, Improving Through Feedback, and Winning Endorsements. Across each strategy, rankings supported the qualitative data findings in feasibility through shared experiences already taking place that can be adopted, through impact in the ability to increase DEI through specified strategies, and through priority rankings in comparison to the other strategies. No divergence was found across quantitative and qualitative data findings.

**Table 4.** Joint display of quantitative, qualitative, and mixed methods

Strategy	Quantitative Findings - Rankings	Qualitative Findings*	Mixed Methods Comparison
Prioritizing Representation	Feasibility – 1	It’s not something that we just pay lip service to, but it’s front and center pretty much everything that we do the communities we serve the faculty that were accrued. And that includes all of our recruitment efforts for scholars in the program. Representation goes beyond that though. We have to think about ideas… One idea is to have a diversity advocate, community partners, which is something that we focus on very intensely within our program to give us feedback on our, our program, accessible mentors and role model models are critically important and then recruitment across the translational perspective. A holistic review is important and department admissions could mirror that.	Convergence: Similarity was found in prioritizing representation as being feasible through shared examples of how it is actively being done, has high impact as characterized as being at the center of communities being served, and is a high priority by discussing partners and advocates at every area of influence to further prioritize representation.
	Impact – 1		
	Priority – 1		
Building Partnerships	Feasibility – 2	We also think about building partners in partnerships in terms of internal recruitment for new faculty that might be looking to become scholars on our advisory boards. We look for a diverse advisory, and we asked specifically for comments on our diversity goals and our perspectives on those goals. And then think about within the institution, are there ways to develop initiatives with the administration to enhance and increase diversity among the programs? We do a lot with the community and bringing research partnerships in liaisons into our working groups and our task force, so that we can always receive feedback and try to act on that feedback.	Convergence: Similarity was found across feasibility, impact, and priority as experiences building both internal and external partnership are shared and the role of community liaisons.
	Impact – 3		
	Priority – 2		
Making it Personal	Feasibility – 3	We think it’s important to have multiple “on ramps” and to build in second chances for those who might have missed the earlier, perhaps most usual, on ramps to research career development. You do not need to BE diverse to be effective in advocating and implementing effective programs. AAMC meeting this week, I listened to the stories behind black doctor’s CVs. so powerful. African American male who was an orphan or another whose father was shot and killed. The struggles that they had to get into medical schools because they didn’t fit the “profile.” This really is about recognizing that, you know, they’re all at our KL2 program and how they get there and where they may be going, can be very different.	Convergence: Similarity was found in rankings for “Making it Personal” as described across qualitative data sources in creating multiple avenues for career development, the importance of the lived experience for scholars, and the role that all mentors can play in supporting this strategy regardless of background.
	Impact – 2		
	Priority – 3		
Designing Program Structure	Feasibility – 4	Thinking about the structures and processes…as you look at your application process, some of the things discussed were really thinking about opportunities for folks to highlight their strengths. That can be in the application process. Some programs actually also will include interviews. And what are the markers of potential of the potential for success? We often think about things like previous grants or papers but are there really other ways that people can show where they’re going in ways that are important for consideration in the selection process. And then as we focus on the needs and goals, really thinking again about how we support diversity throughout in our curriculum meetings with individual scholars. For our TL1, we have a track in CTS in the graduate school that results in a PhD. This has proved very population with outstanding URM students (>50% of the matriculants are URMs). We think that this in part reflects that many are interested in more late-stage translation that can directly impact communities, among other factors. Include cultural competence as part of curriculum.	Convergence: Similarity in rankings and qualitative data was seen, namely as the qualitative data foregrounds structural changes needed, often most challenging and time intensive to change. These challenges are reflective of the rankings.
	Impact – 4		
	Priority – 4		
Improving Through Feedback	Feasibility – 5	You want to obtain feedback from all phases of the program from the recruitment phase all the way through to your alumni after they’ve completed your program feedback should be bi-directional. So to and from the scholars to inform the program directors and people involved in the program and feedback can be either formal or informal or one example that we took from the publication was an institution devotes, the first 15 minutes of their group meetings to open discussion you know, before they get to the formal part. I am not the TL1 fellows’ official research mentor so I try to provide a space where they can safely speak about inclusion and diversity issues and any problems they are having in this regard with their research team. We have added a DEI position to our education leadership. one of his roles is a safe space for students. most common conversations focus on supporting students experiencing microaggressions	Convergence: Similarity is seen across rankings in quantitative polling results and the qualitative data sources, even though ranking from polling data was relatively low. Specifically, across the qualitative data sources experiences are shared of what has and is being done to solicit feedback formally and informally.
	Impact – 6		
	Priority – 6		
Winning Endorsement	Feasibility – 6	Who are your program champions? Hopefully they are you as program directors. But how about your institution’s communications office are they promoting your program and are they promoting your program to, you know, the right stakeholders? How about your current and former scholars? These are likely going to be your best salespeople especially to people within their own discipline people with, from their own gender and from their own racial and ethnic backgrounds. So if you’ve had a good experience or your scholars had good experience, your trainees had a good experience, your master’s student has a good experience and they can tell their peers about it.	Convergence: Similarity across data sources is found as qualitative data sources represent the need to engage stakeholders and former scholars to endorse programs. Depending on the past experiences of former diverse scholars, this may require time for bridging.
	Impact – 5		
	Priority – 5		

*Response presented as written.

Acronyms Defined: AAMC [Association of American Medical Colleges]; BIPOC [Black Indigenous People of Color]; CTS [Clinical and Translational Science]; CTSA [Clinical and Translational Science Awards]; CV [Curriculum Vitae]; DEI [Diversity Equity and Inclusion]; IDP [Individual Development Plan]; KL2 [Mentored Career Development Award]; RCMI [Research Centers in Minority Institutions]; TL1 [Clinical and Translational Science Fellowship]; URM [Underrepresented Minority].

## Discussion

Overall, this mixed methods analysis shows that across a nationally representative sample of CTSA hubs, rankings of the six key strategies as developed and outlined by CLIC are feasible, impactful, and a priority for enhancing diversity and inclusion in Translational Science training programs. Findings from this mixed methods analysis provide robust support for prioritizing the representation of trainees, mentees, and educators as a number one strategy to focus on in training programs. Specifically, the rankings of this strategy can be operationalized through the integration of community representation, diversity advocates, and adopting a holistic approach to recruiting a diverse cadre of scholars into translational science training programs at the national level across CTSAs. This holistic approach includes not only identifying diversity advocates and having community representation, but emphasizing the hiring, promotion, and retention of individuals representing diversity. In addition, creating accessibility to mentors and role models within training programs are also listed as key areas to prioritize representation.

This analysis also shows that building partnerships is a high-priority strategy for training programs. This includes the need for internal and external partnerships through liaisons, task forces, and working groups designed to build support for enhancing DEI within training programs. Finally, rankings for making it personal, designing program structure, and winning endorsements were all supported by the convergence data analysis.

Across the country, CTSA programs are prioritizing efforts to increase DEI in the scientific workforce for enhancing diverse representation across the scientific community. To effectively turn the dial and increase racial and ethnic minority faculty representation, operationalization of identified strategies is needed [12]. These findings add specific support for strategizing representation in training programs as a key area of focus for CTSA programs in support of DEI efforts.

Importantly, the next steps to foster DEI include the need for institutions to apply the evidence base to redesign recruitment, training, and retention structures to support the development and training of diverse scholars across the translational sciences. Important contextual understanding for these efforts includes the consideration of individual circumstances and recognition that URM does not equate to disadvantaged. Applying holistic and system wide efforts to enhance DEI should be approached with full representation in the development and implementation, allowing for modification of the environment to support scholars.

### Limitations

While this study is strengthened by its mixed methods design, there are some key limitations that should be mentioned. First, the fact that quantitative data was based on polled responses limited detailed statistical analyses. Second, although there was broad representation from CTSA hubs, the study did not use a sampling framework to achieve a nationally representative sample. However, the results reflect broad representation from 56 of 64 hubs, which is meaningful. Third, the quantitative sample was relatively small, but for a mixed methods study, the data were sufficient to make a meaningful inference. Finally, using the CLIC From Insights To Action framework as a starting point may have limited introduction of new themes; however, this is consistent with thematic analysis and the intent of the study.

## Conclusions

Translational Science training programs are well positioned to be at the forefront of training the next cadre of diverse leaders in the national health sector. This can be achieved by focusing on prioritizing representation, building partnerships internally and externally to home institutions, making training and development personal throughout the education and training experiences of scholars, redesigning program structures to support and enhance DEI, and having winning endorsement from past and present scholars as well as advocates. Leveraging these findings, the paradigm for workforce diversity and representation within the scientific community can shift, producing more equitable systems of training and healthcare at the population level.
